# Utility of ^18^F-fluoro-deoxyglucose emission tomography/computed tomography fusion imaging (^18^F-FDG PET/CT) in combination with ultrasonography for axillary staging in primary breast cancer

**DOI:** 10.1186/1471-2407-8-165

**Published:** 2008-06-09

**Authors:** Shigeto Ueda, Hitoshi Tsuda, Hideki Asakawa, Jiro Omata, Kazuhiko Fukatsu, Nobuo Kondo, Tadaharu Kondo, Yukihiro Hama, Katsumi Tamura, Jiro Ishida, Yoshiyuki Abe, Hidetaka Mochizuki

**Affiliations:** 1 Department of Surgery, National Defense Medical College, 3-2 Namiki, Tokorozawa, Saitama 359-8513, Japan; 2 Department of Basic Pathology, National Defense Medical College, 3-2 Namiki, Tokorozawa, Saitama 359-8513, Japan; 3 Department of Basic Traumatology, National Defense Medical College, 3-2 Namiki, Tokorozawa, Saitama 359-8513, Japan; 4 Department of Oral Biochemistry, Ashahi University School of Dentistry, 1851 Hozumi, Mizuho, Gifu, 501-0296, Japan; 5 Section of Radiology, National Defense Medical College Hospital, 3-2 Namiki, Tokorozawa, Saitama 359-8513, Japan; 6 Tokorozawa PET Diagnostic Imaging Clinic, 7-5 Higashi-Sumiyoshi, Tokorozawa, Saitama 359-1124, Japan

## Abstract

**Background:**

Accurate evaluation of axillary lymph node (ALN) involvement is mandatory before treatment of primary breast cancer. The aim of this study is to compare preoperative diagnostic accuracy between positron emission tomography/computed tomography with ^18^F-fluorodeoxyglucose (^18^F-FDG PET/CT) and axillary ultrasonography (AUS) for detecting ALN metastasis in patients having operable breast cancer, and to assess the clinical management of axillary ^18^F-FDG PET/CT for therapeutic indication of sentinel node biopsy (SNB) and preoperative systemic chemotherapy (PSC).

**Methods:**

One hundred eighty-three patients with primary operable breast cancer were recruited. All patients underwent ^18^F-FDG PET/CT and AUS followed by SNB and/or ALN dissection (ALND). Using ^18^F-FDG PET/CT, we studied both a visual assessment of ^18^F-FDG uptake and standardized uptake value (SUV) for axillary staging.

**Results:**

In a visual assessment of ^18^F-FDG PET/CT, the diagnostic accuracy of ALN metastasis was 83% with 58% in sensitivity and 95% in specificity, and when cut-off point of SUV was set at 1.8, sensitivity, specificity, and accuracy were 36, 100, and 79%, respectively. On the other hand, the diagnostic accuracy of AUS was 85% with 54% in sensitivity and 99% in specificity. By the combination of ^18^F-FDG PET/CT and AUS to the axilla, the sensitivity, specificity, and accuracy were 64, 94, and 85%, respectively. If either ^18^F-FDG PET uptake or AUS was positive in allixa, the probability of axillary metastasis was high; 50% (6 of 12) in ^18^F-FDG PET uptake only, 80% (4 of 5) in AUS positive only, and 100% (28 of 28) in dual positive. By the combination of AUS and ^18^F-FDG PET/CT, candidates of SNB were more appropriately selected. The axillary ^18^F-FDG uptake was correlated with the maximum size and nuclear grade of metastatic foci (p = 0.006 and p = 0.03).

**Conclusion:**

The diagnostic accuracy of ^18^F-FDG PET/CT was shown to be nearly equal to ultrasound, and considering their limited sensitivities, the high radiation exposure by ^18^F-FDG PET/CT and also costs of the examination, it is likely that AUS will be more cost-effective in detecting massive axillary tumor burden. However, when we cannot judge the axillary staging using AUS alone, metabolic approach of ^18^F-FDG PET/CT for axillary staging would enable us a much more confident diagnosis.

## Background

Axillary lymph node (ALN) status is an important predictor regarding recurrence and survival of patients having primary breast cancer. Recently, sentinel node biopsy (SNB) has been introduced as a minimally invasive procedure to evaluate ALN status [1].

Accurate evaluation of ALN involvement is mandatory before treatment of primary breast cancer by following reasons; (1) ALN status is related to staging of disease and patients prognosis. (2) SNB can be beneficial for the patients to whom the presence of ALN involvement is not preoperatively detectable. They can avoid ALN dissection (ALND) when metastatic foci in sentinel nodes (SNs) are absent. (3) The status of ALN might influence on the decision of primary systemic chemotherapy (PSC). Patients with involved ALNs can be a candidate for PSC.

In our institute, we have carried out the identification of SNs using tin-colloid radioisotope technique [2]. SNs cannot be detected in patients having a massive ALN involvement because of poor uptake of radiotracer in SNs mostly replaced by tumor. Therefore, clinically node-positive patients are not candidates for SNB. Axillary ultrasonography (AUS) has been the most easy-applicable imaging tool for clinical staging of ALN status in patients having primary breast cancer [3,4].

We have used AUS to identify breast cancer patients who were eligible for optimal SNB [5]. Although AUS is not complete for the accurate determination of axillary nodal status, this tool is particularly sensitive for selecting patients with massive tumor burden. Actually, in a series of patients who were judged as candidates for SNB by AUS only, we reported that diagnostic accuracy of ALN status could be achieved 98.6%[5]. Positron emission tomography with ^18^F-fluorodeoxyglucose (^18^F-FDG PET) is expected to be a non-invasive approach to evaluate patients having ALN involvement. Recent studies using ^18^F-FDG PET alone have shown low sensitivity, acceptable specificity, and acceptable positive predictive values in detection of ALN involvement [6-9]. In a prospective multicenter study in the U.S.A, diagnostic performance of ^18^F-FDG PET in axillary staging showed relatively low sensitivity (mean 61%, ranging from 54% to 67%), and high specificity (mean 80%, ranging from 79 to 81%), when abnormal axillary focus was considered positive. In the same study, the ^18^F-FDG PET in axillary staging had a lower sensitivity of 32% and a higher positive predictive value of 90%, when the cut-off of standerd uptake value (SUV) 1.8 or greater [10]. Veronesi et al also reported that the sensitivity of ^18^F-FDG PET for detecting ALN involvement was 37%, but specificity and positive predictive value were 96% and 88%, respectively, at the threshold of SUV 1.2 [11].

Recently, fusion imaging systems combining ^18^F-FDG PET and computed tomography (^18^F-FDG PET/CT) have come to be applied in breast oncology. ^18^F-FDG PET/CT can visualize anatomical location of the hypermetabolic cancer lesions better than ^18^F-FDG PET or CT alone [12,13]. Therefore, the application of ^18^F-FDG PET/CT might be very informative for detecting both regional lymph node involvement and distant metastasis.

We studied both a visual assessment of ^18^F-FDG uptake and SUV for axillary staging, and compared the diagnostic accuracy between preoperative ^18^F-FDG PET/CT and preoperative ultrasound in detecting ALN metastasis. We discussed the clinical management of the combonation of axillary ^18^F-FDG PET/CT and AUS for the selection of proper candidates of SNB and PSC.

## Methods

### Patients

The study was done in accordance with the ethical principles of the Declaration of Helsinki and was approved by the institutional review board in the National Defense Medical College (NDMC). Informed consents were obtained from all patients with regard to ^18^F-FDG PET/CT examination and the entry into the present study.

This prospective study enrolled a series of 183 patients having primary breast cancer proven by core needle biopsy at the National Defense Medical College Hospital from April 2005 through August 2007. For axillary staging, all patients underwent both ^18^F-FDG PET/CT and ultrasonography within 5 weeks before surgery. Patients with diabetes mellitus or pregnancy, those who underwent primary systemic therapy, those who underwent excisional biopsy were excluded. During the entry period, we experienced 15 patients who were diagnosed by ^18^F-FDG PET/CT to have breast cancer with distant metastases, comprising four distant lymph node metastases, nine bone metastases, and two lung metastases. Patients having distant metastases were also ineligible for this study.

### Axillary ultrasound examination

AUS was performed using ProsoundII SSD 6500 (Aloka, Tokyo, Japan) employing a 10-MHz linear array transducer. AUS criteria in our institute were described previously [5]. In brief, homogeneously hypoechoic lymph nodes with diameters of 10 mm or more and in oval or round shape were defined as AUS-positive and were considered to be potentially extensive nodal involvement. Lymph nodes with central hyperechoic area and/or with diameter of less than 10 mm, defined as AUS-negative were considered to be clinically node-negative. One experienced ultrasonographer (T.K) performed axillary examination of operable patients. At least two breast surgeons discussed and determined AUS status together with the ultrasonographer in weekly conference.

### Surgery and Sentinel node biopsy procedure

The 183 patients underwent mastectomy or breast-conserving surgery with SNB and/or ALND. SNB was performed using the procedure described previously [2]. According to SNB protocol in our institute, patients having AUS negativity were eligible for SNB and were optionally performed SNB after the acquisition of informed consent. For patients having AUS positivity or those who rejected SNB, ALND was performed.

SNs were intraoperatively examined histopathologically. Biopsied SNs were cut into 2-mm-thick slices, and histopathological sections were made from each slice. These sections were stained with hematoxylin and eosin, and at least two pathologists examined the sections. All patients having SN metastasis recieved ALND. If SNs were free of cancer cells, ALND was omitted.

### ^18^F-FDG PET/CT and quantification of ^18^F-FDG uptake in axilla

All patients received ^18^F-FDG PET/CT scans (Biograph LSO Emotion, 3D model, Siemens, Germany) at Tokorozawa PET Diagnostic Imaging Clinic (Tokorozawa, Japan). Blood glucose level was measured in each patient and did not exceed 120 mg/dl.

Patients fasted at least 4 hours before ^18^F-FDG PET study. One hour after intravenous administration of 3.7 Mbq/kg ^18^F-FDG, a transmission scan using CT (SOMATO Emotion, 16-slice configuration, pitch 1.83, Siemens, Germany) for attenuation correction and anatomical imaging was acquired for 90 sec. IV contrast was not administered to patients for the CT portion of the ^18^F-FDG PET/CT.

Back projection image was obtained after Gaussian filter was applied. The spatial resolution of the reconstructed images was 6.0–7.0 mm in cranio-caudal, 6.3–7.1 mm in right-left and 6.3–7.1 mm in anterior-posterior directions.

A regions of interest (ROI) was placed in the axillary lesion, including the highest uptake area (circle ROI, 2 cm in diameter), and SUV maximum in the ROI was calculated. The SUV was decay-corrected tissue activity divided by the injected dose per patient body, and was calculated using the following formula: SUV = activity in region of interest/decay factor of F-18 (MBq/ml)/injected dose (MBq/kg body weight).

CT images were also available for evaluation. Visual assessment of ^18^F-FDG uptake was carried out by at least two experienced nuclear medicine radiologists, and abnormal axillary uptake greater than background activity was interpreted as suspicious nodal involvement. Semiquantitative measurement of SUV was done on any axillary focus with abnormal uptake.

### Determination of the optimal SUV cut-off points

To determine the optimal SUV cut-off point, the tentative SUV cut-off point was established, ranging from 0.8 to 3.0 with 0.2 to 0.3 increments. SUV of the cut-off point or greater was defined as positive and SUV less than the point was defined as negative. The SUV of 0.4 is the lowest limit of visible uptake of ^18^F-FDG. Based on each SUV cut-off point, the diagnostic accuracy of ^18^F-FDG PET/CT (positive or negative) was evaluated by means of sensitivity, specificity, positive predictive value (PPV), and negative predictive value (NPV).

### Histopathological study

The number, the maximum size, and nuclear grade of involved ALNs were histopathologically examined.

### Statistical analysis

Statistical analysis was performed using Statview 5.0 version (SAS Institute Inc). As univariate analysis, Mann-Whitney *U *test and chi square test were used to establish the correlation between clinicopathological variables and axillary SUV. *P *value of 0.05 or less was defined as statistical significance.

## Results

### Patient characteristics

Patient age, pathological T factor, histological type, nuclear grade, hormonal receptor status and c-erbB2 status of the primary tumors, and SUV of the primary tumors and axillary uptake are listed in Table 1, ALN involvement was histopathologically detected in 59 (32%) of 183 patients.

**Table 1:** Patient characteristics

Variables		Number 183	% 100
Age	mean [range]	57 [32–81]	
	<45	33	18
	45 ≦	150	82
pT-stage	pTis	10	5
	pT1	91	50
	pT2	68	37
	pT3	14	8
Histology	DCIS	9	5
	IDC	158	86
	ILC	9	5
	Apocrine	2	1
	Mucinous	2	1
	Squamoid	1	1
	Paget	2	1
Nuclear grade	1	59	32
	2	51	28
	3	69	38
	Not graded	4	2
Nodal metastasis	negative	124	68
	positive	59	32
Estrogen receptor (ER)	10%>	44	24
	10% ≦	139	76
Progesterone receptor(PgR)	10%>	63	34
	10% ≦	120	66
c-erbB-2 (HER2)	0 to 2+	152	83
	3+/FISH Amp	28	15
	unknown	3	2
Primary axillary approach	Ax dissection	58	32
	SNB	125	68
SUV of the primary tumor	mean [range]	4.3 [0.9–17.8]	
SUV of axillary uptake*	mean [range]	3.0 [0.4–11.3]	

DCIS, ductal carcinoma in situ; IDC, invasive ductal carcinoma; ILC, invasive lobular carcinoma; Mucinous, mucinous carcinoma; Apocrine, apocrine. carcinoma; Squamoind, Squamoid carcinoma; Paget, Paget's disease; FISH Amp, FISH Amplication; Ax, axillary; SNB, sentinel node biopsy; SUV, Standardized Uptake Value; *Visible uptake of 18F-FDG

The mean SUV of the primary tumor in the 183 patients was 4.3, ranging from 0.9 to 17.8, and the mean SUV of the axilla was 3, ranging 0.4 to 11.3. The SUVs of 0.4 and 11.3 were the lowest point and the highest point to visualize ^18^F-FDG uptake, respectively. The mean interval between ^18^F-FDG PET/CT and surgery was 29 days. One hundred twenty-five (68%) of the 183 patients underwent initial SNB. One hundred twenty-four (99%) of the 125 patients were successfully performed SNB. The number of removed SNs per patient was 2.4 on an average. Metastases to SNs were positive in 24 (19%) patients, and all of these patients underwent ALND. Metastases to the SNs were negative in 100 (81%) patients, and none of them recieved further axillary surgery. Other 58 (32%) patients underwent ALND without SNB.

### Diagnostic performance of ^18^F-FDG PET/CT and ultrasonography in axillary staging

Diagnostic performance for detecting axillary involvement was compared between ^18^F-FDG PET/CT and ultrasonography (Table 2).

**Table 2:** Diagnostic performance of ^18 ^F-FDG PET/CT and ultrasonography in axillary staging

^18^F-FDG uptake	TP	TN	FP	FN	Sensitivity (%)	Specificity (%)	PPV (%)	NPV (%)	Accuracy (%)
Visual assessment	34	118	6	25	57.6	95.2	85	82.5	83.1

SUV cutoff point									
0.8	30	118	6	29	50.8	95.2	83.3	80.3	80.9
1.3	24	122	2	35	40.7	98.4	92.3	77.7	79.8
1.5	21	123	1	38	35.6	99.2	95.5	76.4	78.7
1.8	21	124	0	38	35.6	100	100	76.5	79.2
2	20	124	0	39	33.9	100	100	76.1	78.7
3	16	124	0	43	27.1	100	100	74.3	76.5

AUS	32	123	1	27	54.2	99.2	97	82	84.7

Visual assessment of ^18^F-FDG uptake Combined with AUS	38	117	7	21	64.4	94.4	84.4	84.8	84.7

AUS, Axillary ultrasonography; TP, True positive; TN, True negative; FP, False positive; FN, False negative; PPV, Positive predictive value; NPV, Negative Predictive value;

By visual assessment of ^18^F-FDG PET/CT, axillary uptake was positive in 40 (22%) patients and negative in 143 (78%) patients. Of these 40 axillary-positive patients, 34 (85%) were truely positive, whereas 6 (15%) were false positive. Of the 143 axillary- negative patients, 118 (83%) patients were truely negative, whereas 25 (17%) patients were false negative. Sensitivity, specificity, PPV, NPV, and accuracy of visual assessment of ^18^F-FDG PET/CT were 58, 95, 85, 83, and 83%, respectively.

The accuracy of diagnosis of ^18^F-FDG PET/CT was compared among various SUV cut-off points ranging from 0.8 to 3.0, using entire data set of 183 patients.

When a SUV cut-off points were set from 0.8 up to 1.8, specificity increased from 95% to 100%, but sensitivity decreased from 51% to 36%. As SUV increased over 1.8, specificity of 100% did not vary, but sensitivity further decreased. When the SUV was 1.8, PPV, NPV and accuracy were 100%, 77%, and 79%, respectively. Therefore, the SUV of 1.8 achieved excellent specificity and PPV, but low sensitivity in comparison with visual assessment.

Ultrasonography detected 33 (18%) AUS-positive patients and 150 (82%) AUS-negative patients. Of the 33 AUS-positive patients, thirty-two patients (97%) were truely positive, and one patient (3%) was false-positive. Of the 150 AUS-negative patients, 123 (82%) were truely negative, whereas 27 (18%) were false-negative. Sensitivity, specificity, PPV, NPV, and accuracy were 54, 99, 97, 82, and 85%, respectively.

Combined with visual assessment of ^18^F-FDG uptake and AUS, 138 (75%) patients with double-negative ^18^F-FDG uptake and AUS were considered to be nodal negative, and 45 (25%) patients with positive finding in the visual assessment of ^18^F-FDG uptake and/or AUS were considered to be nodal positive. Sensitivity, specificity, PPV, NPV, and accuracy of the combination were 64, 94, 84, 85, and 85%, respectively.

### Feasibility of SNB for patients having negative AUS

Of the 150 patients having negative AUS, 125 (83%) consented and underwent SNB. Table 3 shows diagnostic performance of SNB in axillary staging in AUS-negative patients. SNB identification rate was 99.2% (124 of 125 patients). Twenty five patients (20%) had axillary nodal metastasis in permanent pathology. Intraoperative pathological diagnosis of metastasis in SNB was accurately performed in 123 (99%) of 124 patients. One patient (1%) was false negative by frozen section intraoperatively, but a micrometastatic deposit was postoperatively detected in one of sentinel nodes by permanent histology. With regard to intraoperative pathological diagnosis of metastasis in SNB, sensitivity, specificity, PPV, NPV and overall accuracy were 96, 100, 100, 99, and 99% respectively in Table 3A.

**Table 3:** Diagnostic performance of SNB for axillary staging in AUS-negative patients

			No. of patients	
			
			Permanent histopathology	
Intraoperative frozen Histopathology		Total	Metastasis positive	Metastasis negative

A.	Total (n = 124)			
	Metastasis positive	24	24	0
	Metastasis negative	100	1	99

	Total	124	25	99

B.	^18^F-FDG uptake positive (n = 12)			
	Metastasis positive	5	5	0
	Metastasis negative	7	1	6

	Total	12	6	6

C.	^18^F-FDG uptake negative (n = 112)			
	Metastasis positive	19	19	0
	Metastasis negative	93	0	93

	Total	112	19	93

Note; SNB, sentinel node biopsy; AUS, axillary ultrasound; No., Number; SNB identification rate 99.2% (124 of 125 cases)

Sensitivity, specificity, positive predictive value (PPV), negative predictive value (NPN), and accuracy overall were 96, 100, 100, 99, and 99%, respectively in A. Sensitivity, specificity, PPV, NPV, and overall accuracy were 83, 100, 100, 86, and 92%, respectively in B.

Sensitivity, specificity, PPV, NPV, and overall accuracy were 100, 100, 100, 100, and 100%, respectively in C.

In the 12 AUS-negative but ^18^F-FDG uptake positive patients who consented and recieved SNB, 6 (50%) had ALN involvement. One (8%) patient was false negative. With regard to intraoperative pathological diagnosis of SLN, sensitivity, specificity, PPV, NPV, and overall accuracy were 83, 100, 100, 86, and 92%, respectively in Table 3B.

In the 112 AUS-negative and ^18^F-FDG uptake negative patients, who consented and underwent SNB, 19 (17%) had ALN involvement. With regard to intraoperative pathological diagnosis of SNB, sensitivity, specificity, PPV, NPV, and overall accuracy were all 100% in Table 3C.

### Axillary nodal clinicopathological factors correlated with ^18^F-FDG uptake

Table 4 shows correlation of axillary ^18^F-FDG uptake with nodal clinicopathological factors of 59 patients having ALN involvement. The maximum size and nuclear grade of involved ALN were significantly correlated with ^18^F-FDG uptake at SUV cut-off point 1.8 (*p *= 0.006 and 0.03, respectively). The number of involved ALNs was not correlated with ^18^F-FDG uptake at SUV cut-off point 1.8 (*p *= 0.15).

**Table 4:** Axillary nodal clinicopathological factors correlated with ^18 ^F-FDG uptake

Clinicopathological factors	SUV cut-off 1.8	No. of pts	Average	SD	*p*-value
No. of involved ALNs	Low	38	3.6	5	0.15
	High	21	6.9	7	
The maximum size (mm)	Low	38	8.6	6.4	0.006
	High	21	14.6	7.9	

	SUV	No. of pts	Grade	No. ofpts	*p*-value

Nuclear grade	Low	38	Grade1/2	27	0.03
			Grade3	11	
	High	21	Grade1/2	9	
			Grade3	12	

SUV, Standardized uptake value; No, Number; pts, patients; SD, Standard derivation

### Categories of ^18^F-FDG PET/CT combined with ultrasound for indications of ALND and PSC

Table 5 indicates four categories which were divided by clinical findings of ^18^F-FDG PET/CT and AUS to the axilla. We used a visual assessment of ^18^F-FDG uptake which proved to be a reproducible method and have acceptable sensitivity and specificity [6]. Of all 183 patients, 138 (75%) patients who had negative AUS with negative axillary ^18^F-FDG uptake were classified as category 1. Frequency of ALNs involvement was only 21 (15%), 12 (57%) of these 21 patients had single involved ALNs. The maximum size of involved ALNs was 10 mm or smaller in 17 (81%) of 21 patients. The nuclear grade of involved ALNs was grade 1 or 2 in 16 (76%), and grade 3 in only 5 (24%).

**Table 5:** Categories of ^18 ^F-FDG PET/CT combined with ultrasonography for indications of ALND/PSC

Category	1	2	3	4
AUS	-	-	+	+
^18^F-FDG-uptake	-	+	-	+

No. of involved ALNs				
0	117 (85)	6 (50)	1 (20)	0 (0)
1	12 (9)	5 (42)	2 (40)	9 (32)
2 ≦ Involved ALNs ≦ 5	6 (4)	1 (8)	1 (20)	9 (32)
6 ≦	3 (2)	0 (0)	1 (20)	10 (36)

The maximum size of involved ALNs				
≦ 5 mm	10(48)	5 (83)	0 (0)	0 (0)
5 mm < metastasis < 10 mm	7(33)	1 (17)	2 (50)	6 (21)
10 mm ≦	4(19)	0 (0)	2 (50)	22(79)

Nuclear grade of involved ALNs				
Grade 1 and 2	16(76)	3 (50)	4 (100)	13(46)
Grade 3	5(24)	3 (50)	0 (0)	15(54)

Frequency of involved ALNs	15%	50%	80%	100%

Indications of ALND/PSC	SNB	FNAC/Bx is needed	FNAC/Bx is needed	Acceptable

Total	138 (100)	12 (100)	5 (100)	28 (100)

AUS, Axillary ultrasound; ALN, Axillary lymph node; ALND, ALN dissection; PSC, Primary systemic chemotherapy; No., Number; pts, patients; FNAC, Fine needle aspiration

Twenteen (7%) patients who had negative AUS but positive axillary ^18^F-FDG uptake were classified as category 2. In category 2, ALNs involvement was detected in 6 (50%) of 12. A single ALN involvement was present in 5 (83%) of these 6. All 6 patients had ALNs involvement with the maximum size of less than 10 mm in category 2. Nuclear grade was 1 or 2 in 3 (50%) patients, whereas nuclear grade was 3 in 2 (50%).

Five (3%) patients who had positive AUS but negative axillary ^18^F-FDG uptake were classified as category 3. Twenty-eight (15%) patients who had double-positive nodal status of AUS and ^18^F-FDG uptake were classified as category 4. Four (80%) of 5 patients in category 3 had ALNs involvement and all patients in category 4 had ALNs involvement. Especially 2-or-more involved ALNs were 2 (50%) of 4 cases in category 3, and 19 (68%) of 28 cases in category 4. The maximum size of involved ALNs was 10 mm or more in 2 (50%) of 4 cases in category 3 and in 22 (79%) of 24 cases in category 4.

Nuclear grade was 1 or 2 in all 4 (100%) cases in category 3, whereas nuclear grade was 1 or 2 in 13 (46%) of 28 cases in category 4.

### Diagnostic performance of SNB in ^18^F-FDG-positive and AUS-negative patients

Table 6 shows diagnostic performance of SNB for axillary staging in ^18^F-FDG-positive and AUS-negative patients of category 2. Six (50%) of 12 patients had involved SNs and others (50%) had no involved SNs in spite of ^18^F-FDG uptake. No metastases were found in non-SNs in all patients that had involved SNs and received aubsequent axillary dissection.

**Table 6:** Diagnostic performance of SNB for axillary staging in ^18 ^F-FDG-positive and AUS-negative patients of category 2

Patients	SUV	Involved SNs/resected SNs	Involved non-SNs/resected non-SNs	Ax dissection
1	8.1	1/1	0/10	Performed
2	2.5	1/1	0/17	Performed
3	2.4	1/2	0/12	Performed
4	1.4	1/3	0/21	performed
5	1.3	4/4	0/12	performed
6	0.7	1/4	0/6	performed
7	1.5	0/1	-	not performed
8	1	0/4	-	not performed
9	1	0/1	0/1	not performed
10	1	0/4	0/1	not performed
11	0.9	0/1	0/2	not performed
12	0.9	0/4	0/1	not performed

AUS, Axillary ultrasound;SUV, Standardized uptake value; SNs, Sentinel nodes, Ax, Axillary

## Discussion

### Visual assessment of ^18^F-FDG PET/CT for the axillary staging

In visual assessment of ^18^F-FDG PET/CT to the axilla, we demonstrated that diagnostic accuracy of ^18^F-FDG PET/CT was almost equivalent to that of AUS for detecting of ALN involvement in patients with primary breast cancer. Visual assessment of ^18^F-FDG uptake to the axilla achieved higher sensitivity than AUS, and the specificity and PPV of ^18^F-FDG PET/CT were acceptably high, 95%, and 85%, respectively.

There were 40 (22%) of 183 patients having axillary uptake of ^18^F-FDG. Six (15%) of these patients had no metastasis of ALNs. The reason of these false positive for the ^18^F-FDG uptake is not known, but reactive lymphadenopathy caused by breast biopsy would lead to false positive results [9,14].

AUS showed limited sensitivity equal to ^18^F-FDG PET/CT for detecting ALN involvement, and showed almost perfect specificity and PPV. According to diagnostic performance of axillary ultrasonography, the present results showed higher outcome than others' previous studies [15-17]. In our previous study, AUS was performed using an SSD-650CL (Aloka, Tokyo, Japan), an old model of SSD-6500, and indicated sensitivity, specificity, PPV, and overall accuracy of 45, 97, 92.6, and 75%, respectively [5]. Furthermore an ultrasound specialist performed axillary investigation in the present study. From these reasons, we considered the present results were superior to those in our previous study.

Differences in criteria for judgment of axillary status or in the type of ultrasound device might have given rise to such inconsistency. Furthermore, although ultrasonography is less-invasive and relatively easy to apply, experienced skills are required to judge AUS-positive nodes. We sometimes wavered in our judgement whether ALNs were positive or not when ultrasound image of lymph nodes was less than 10 mm in diameter but homogeneously hypoechoic in centric area. From these reasons, AUS alone might be difficult to determine axillary staging.

We classified patients into 4 categories of axillary status according to ^18^F-FDG PET/CT and ultrasonography (Table 5).

Category 1 showed the patients who have AUS-negative lymph nodes without axillary ^18^F-FDG uptake. Fifteen percent of these 138 patients had ALNs involvement. Characteristics of ALN involvement were lesser number, smaller sizes, and lower nuclear grade of metastatic foci (Table 5). For these patients, SNB was successfully performed as shown in Table 3, and SNB is recommended to assess axillary nodal status.

Category 2 and 3 showed the patients having discrepancy between the axillary examinations of AUS and ^18^F-FDG uptake.

Category 2 showed the patients having ^18^F-FDG uptake but negative AUS. Half of these patients have metastatic foci in their axilla. The reason of the discrepancy was related to the fact that metastatic foci of small size (5 mm or less) and/or higher nuclear grade was detected by ^18^F-FDG uptake but were not by AUS.

Category 3 showed the patients having positive AUS without axillary ^18^F-FDG uptake. We found 4 (80%) of 5 patients had ALN involvement. The characteristic of these metastastic foci was lower nuclear grade. This result are in keeping with previous reports [8-10].

The conclusions could not be determined because the number of patients in categories 2 and 3 have been limited, but we could indicate lymph nodes having discrepancy in diagnosis between AUS and axillary ^18^F-FDG uptake were found to be frequently metastasized. When the discrepancy occurred between these two modalities, therefore, we suggest further axillary investigations such as core-needle biopsy, or fine needle aspiration cytology to evaluate precisely axillary nodal status.

We confirmed the positive lymph nodes found by ^18^F-FDG-PET matched the SNB results in all patients of category 2 that had involved SNs and received subsequent axillary dissection (shown in Table 6).

Category 4 showed the patients had double-positive ALNs of AUS and ^18^F-FDG uptake. PPV for detecting ALN involvement was 100%. These patients were recommended to undergo ALND without SLN. In addition, it might be rational to consider that patients having AUS positive nodes and axillary ^18^F-FDG uptake will have PSC without biopsy or fine needle aspiration cytology to the axilla.

### Semiquantitative assessment of ^18^F-FDG PET/CT for the axilla

Table 4 showed higher SUV were significantly correlated with nuclear grade 3 and maximum size of metastatic foci but not with number of involved ALNs. These results also appear to reveal biological significance of axillary ^18^F-FDG accumulated to metastasized cancer cells.

When the cut-off of SUV exceeded 1.8, specificity and PPV of ^18^F-FDG PET/CT were almost 100%, but sensitivity notably decreased to 36% or lower.

From the present results, appropriate determination of the cut-off of SUV appeared possible to evaluate ALN involvement by means of ^18^F-FDG uptake. Especially by setting of cutoff of SUV, we could predict ALN involvement with excellent specificity and PPV. The cut-off of SUV for ALN involvement varies from 1.2 to 2.3 among reports previously published [9,11]. The inter-institutional standardization of the cut-off value-off SUV for ALN evaluation remains to be settled.

Thus, we found that axillary ^18^F-FDG uptake added incremental diagnostic confidence to AUS. Richard L et al reported that ^18^F-FDG PET may have a role in assessing patients with medially or superiorly situated breast cancers that may drain preferentially or exclusively to internal mammary or supraclavicular nodes[10]. A. Gil-Rendo et al also described that an advantage of ^18^F-FDG PET was to be able to detect internal mammary node metastasis, which is often clinically occult and poorly visualized by conventional modality including ultrasonography [8].

We experienced a patient having ^18^F-FDG uptake in a parasternal lymph node in spite of double-negativity in AUS and axillary ^18^F-FDG uptake. The lymph node has been proven to be metastasized by fine-needle aspiration cytology. Another patient who have double-positivity in AUS and axillary ^18^F-FDG uptake, also showed ^18^F-FDG uptake in infraclavicular lymph nodes. These two patients had chosen PSC, having been ineligible for this study protocol.

Thus, we considered the whole-body ^18^F-FDG PET/CT would be informative for imaging investigations for regional nodes involvement as well as distant metastasis [12,18].

## Conclusion

In conclusion, the diagnostic accuracy of visual assessment of ^18^F-FDG PET/CT was almost equivalent to that of AUS in sensitivity, specificity, and overall accuracy. When cut-off of SUV was set at 1.8 or more, specificity and PPV was each 100%. However, there are numerous factors that will influence SUV results and we should take into consideration the limited value of SUV in breast.

To our knowledge, this is the first study to compare between ^18^F-FDG PET/CT and ultrasonography for detecting of ALN involvement.

Considering their limited sensitivities, the high radiation exposure by ^18^F-FDG PET/CT and also costs of the examination, it is likely that AUS will be more cost-effective in detecting massive axillary tumor burden. However, when we cannot judge the axillary staging using AUS alone, metabolic approach of ^18^F-FDG PET/CT for axillary staging would enable us a much more confident diagnosis.

## Abbreviations

ALN: axillary lymph node; ALND: ALN dissection; AUS: axillary ultrasonography; NPV: negative predictive value; ^18^F-FDG PET/CT: positron emission tomography/computed tomography with ^18^F-fluorodeoxyglucose; SNB: sentinel node biopsy; SUV: standardized uptake value; PPV: positive predictive value.

## Competing interests

The authors declare that they have no competing interests.

## Authors' contributions

SU performed the planning, acquisition of data, analysis of data, and writing of the manuscript. HT (pathologist) performed the planning, interpretation of data, and the manuscript in co-operation with SU. HA, JO, and KF (breast surgeons) performed surgery and the statistic analysis. YH, KT, JI, and YA (radiologists) performed the evaluation of tumoral SUV levels and data acquisition. NK (biochemist) performed the statistic analysis. TK (ultrasonographer) carried out axillary assessment. HM participated in its design and coordination in co-operation with SU and HT. All authors read and approved the final manuscript.

## Pre-publication history

The pre-publication history for this paper can be accessed here:



## References

[B1] Aarsvold JN, Alazraki NP (2005). Update on detection of sentinel lymph nodes in patients with breast cancer. Semin Nucl Med.

[B2] Sato K, Uematsu M, Saito T, Ishikawa H, Yamasaki T, Tamaki K, Tamai S, Kusano S, Hiraide H, Mochizuki H (2000). Indications and technique of sentinel lymph node biopsy in breast cancer using 99m-technetium labeled tin colloids. Breast Cancer.

[B3] van Rijk MC, Teertstra HJ, Peterse JL, Nieweg OE, Olmos RA, Hoefnagel CA, Kroon BB (2006). Ultrasonography and fine-needle aspiration cytology in the preoperative evaluation of melanoma patients eligible for sentinel node biopsy. Ann Surg Oncol.

[B4] Nori J, Vanzi E, Bazzocchi M, Bufalini FN, Distante V, Branconi F, Susini T (2007). Role of axillary ultrasound examination in the selection of breast cancer patients for sentinel node biopsy. Am J Surg.

[B5] Sato K, Tamaki K, Tsuda H, Kosuda S, Kusano S, Hiraide H, Mochizuki H (2004). Utility of axillary ultrasound examination to select breast cancer patients suited for optimal sentinel node biopsy. Am J Surg.

[B6] van der Hoeven JJ, Hoekstra OS, Comans EF, Pijpers R, Boom RP, van Geldere D, Meijer S, Lammertsma AA, Teule GJ (2002). Determinants of diagnostic performance of [F-18]fluorodeoxyglucose positron emission tomography for axillary staging in breast cancer. Ann Surg.

[B7] Barranger E, Grahek D, Antoine M, Montravers F, Talbot JN, Uzan S (2003). Evaluation of fluorodeoxyglucose positron emission tomography in the detection of axillary lymph node metastases in patients with early-stage breast cancer. Ann Surg Oncol.

[B8] Gil-Rendo A, Zornoza G, Garcia-Velloso MJ, Regueira FM, Beorlegui C, Cervera M (2006). Fluorodeoxyglucose positron emission tomography with sentinel lymph node biopsy for evaluation of axillary involvement in breast cancer. Br J Surg.

[B9] Chung A, Liou D, Karlan S, Waxman A, Fujimoto K, Hagiike M, Phillips EH (2006). Preoperative FDG-PET for axillary metastases in patients with breast cancer. Arch Surg.

[B10] Wahl RL, Siegel BA, Coleman RE, Gatsonis CG (2004). Prospective multicenter study of axillary nodal staging by positron emission tomography in breast cancer: a report of the staging breast cancer with PET Study Group. J Clin Oncol.

[B11] Veronesi U, De Cicco C, Galimberti V, Fernandez J, Rotmensz N, Viale G, Spano G, Luini A, Intra M, Veronesi P, Berrettini A, Paganelli G (2007). A comparative study on the value of FDG-PET and sentinel node biopsy to identify occult axillary metastases. Ann Oncol.

[B12] Juweid ME, Cheson BD (2006). Positron-emission tomography and assessment of cancer therapy. N Engl J Med.

[B13] Tatsumi M, Cohade C, Mourtzikos KA, Fishman EK, Wahl RL (2006). Initial experience with FDG-PET/CT in the evaluation of breast cancer. Eur J Nucl Med Mol Imaging.

[B14] Rousseau C, Bourbouloux E, Campion L, Fleury N, Bridji B, Chatal JF, Resche I, Campone M (2006). Brown fat in breast cancer patients: analysis of serial (18)F-FDG PET/CT scans. Eur J Nucl Med Mol Imaging.

[B15] Ohta M, Tokuda Y, Saitoh Y, Suzuki Y, Okumura A, Kubota M, Makuuchi H, Tajima T, Yasuda S, Shohtsu A (2000). Comparative efficacy of positron emission tomography and ultrasonography in preoperative evaluation of axillary lymph node metastases in breast cancer. Breast Cancer.

[B16] Hashimoto H, Suzuki M, Oshida M, Nagashima T, Yagata H, Shishikura T, Imanaka N, Nakajima N (2000). Quantitative ultrasound as a predictor of node metastases and prognosis in patients with breast cancer. Breast Cancer.

[B17] Watermann DO, Tempfer CB, Hefler LA, Parat C, Stickeler E (2005). Ultrasound criteria for ductal invasive breast cancer are modified by age, tumor size, and axillary lymph node status. Breast Cancer Res Treat.

[B18] Fueger BJ, Weber WA, Quon A, Crawford TL, Allen-Auerbach MS, Halpern BS, Ratib O, Phelps ME, Czernin J (2005). Performance of 2-deoxy-2-[F-18]fluoro-D-glucose positron emission tomography and integrated PET/CT in restaged breast cancer patients. Mol Imaging Biol.

